# A Soldier Somnambulist

**Published:** 1888-12

**Authors:** 


### A SOLDIER SOMNAMBULIST.

A batallion of the Third Regiment of Marine Infantry, counts among its members a young soldier somnambulist who has been sent to the hospital at Rochefort. In a state of somnambulism this young man responds to all questions which are put to him; he predicts what is to come and sees things at a distance with marvelous clearness.

Here we select among others, these facts, which are attested by a number of witnesses. A key had been hidden, for which a long search was made without any of the searchers being able to discover it. The somnambulist upon being interrogated, indicated where it was to be found.

Another time, two soldiers of his batallion stole the coffee of the ordinary, which they sold to a third person ; more than a hundred kilogrammes having thus disappeared. It was extremely difficult to discover the culprits, and they questioned the somnambulist at a venture, who named and denounced them. The guilty ones were forced to confess and the affair is, to-day, understood. The receiver and the two soldiers were convicted at the next sessions.

Here is still another fact more strange than the foregoing ones : During a state of somnambulence the same subject declared that a detachment of his regiment which had been sent to New Caledonia, had arrived there the 14th of July, and had disembarked at 7 o'clock in the morning. The telegraph was resorted to in order to ascertain the truth of the matter, and found it to be precisely as the somnambulic soldier had stated. —Translated from *La Spiritisme* of September.

